# 16S rRNA Gene Amplicon Sequencing of Microbiota in Polybutylene Succinate Adipate-Packed Denitrification Reactors Used for Water Treatment of Land-Based Recirculating Aquaculture Systems

**DOI:** 10.1128/MRA.01295-19

**Published:** 2019-11-21

**Authors:** Takeshi Yamada, Masako Hamada, Miku Nakagawa, Nobukazu Sato, Akinori Ando, Jun Ogawa, Mari Yasuda, Tomoki Kawagishi

**Affiliations:** a Department of Applied Chemistry and Life Science, Toyohashi University of Technology, Toyohashi, Aichi, Japan; b Salmon and Freshwater Fisheries Research Institute, Hokkaido Research Organization, Eniwa, Hokkaido, Japan; c Division of Applied Life Sciences, Graduate School of Agriculture, Kyoto University, Kyoto, Japan; d Research Unit for Physiological Chemistry, Kyoto University, Kyoto, Japan; e Tsurumi R&D Center, Mitsubishi Chemical Corporation, Yokohama, Kanagawa, Japan; Georgia Institute of Technology

## Abstract

Information on the microbiota in polybutylene succinate adipate (PBSA)-packed denitrification reactors is limited. Here, we provide 439,817 high-quality reads of the 16S rRNA gene sequences of microbiota in PBSA-packed denitrification reactors used for land-based recirculating aquaculture. The predominant microorganisms belonged to the following families: *Nocardiaceae*, *Chitinophagaceae*, *Xanthobacteraceae*, *Burkholderiaceae*, *Rhodocyclaceae*, *Pseudomonadaceae*, *Rhodanobacteraceae*, and *Xanthomonadaceae*.

## ANNOUNCEMENT

Biodegradable plastics act as carriers for microorganism adhesion and as electron donors in denitrification reactors for removing nitrate from land-based recirculating aquaculture systems (RAS) (1–4). Biofilms form around the biodegradable plastics packed in the denitrification reactors and contribute to biological denitrification (3, 4). However, limited information regarding the microbiota adhering to the biodegradable plastics, particularly to polybutylene succinate adipate (PBSA), is available. Here, we provide 16S rRNA gene amplicon profiles of the microbiota attached to PBSA to achieve efficient nitrate removal in PBSA-packed denitrification reactors for water treatment in RAS.

**FIG 1:**
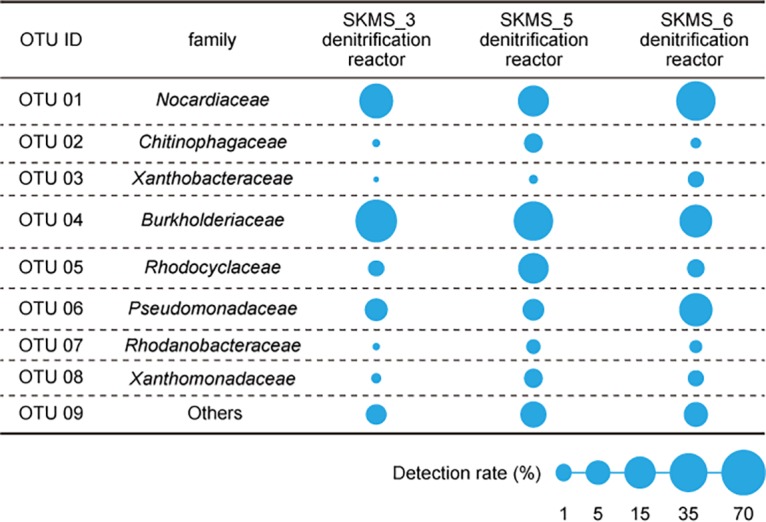
Relative abundances of microbiota in the three PBSA-packed denitrification reactors after 93 days of operation. Relative abundances (>1%) of the microbiota at the family level are displayed in a bubble plot.

PBSA-packed denitrification reactors installed in three RAS tanks (250 liters) (established in Eniwa, Japan, in 2018) discharged breeding water with a nitrate concentration of <60 mg-N·liter^−1^ after 93 days of operation. The three reactors were loaded with PBSA pellets (Bio-PBS; Mitsubishi Chemical Corporation, Tokyo, Japan) and continuously supplied with breeding water (Table 1). Despite the difference in feeding frequency, each RAS tank bred 20 rainbow trout (average weight per fish, 92.5 ± 1.4 g) (Table 1). The sludge samples adhering to PBSA pellets were collected with the pellets from each reactor. DNA from the samples was extracted with a bead-beating method using 1-mm zirconia beads (5) and then purified according to a previous method (5). The V4 region of the 16S rRNA gene was amplified using Blend *Taq* polymerase (Toyobo, Osaka, Japan) and the 515F/806R primer set (6), with the following PCR conditions: 25 cycles of denaturation at 95°C for 30 s, annealing at 50°C for 30 s, and extension at 72°C for 30 s. Sequencing libraries of PCR products for each sample were prepared with three biological replicates, and sequences were determined using a MiSeq instrument and a MiSeq reagent kit v2 (2 × 300 bp; Illumina, San Diego, CA, USA) at the Bioengineering Lab. Co., Ltd. (Kanagawa, Japan). Low-quality sequences were removed using Sickle v1.33 with the following parameters: -q 20 and -l 40 (7). High-quality paired-end reads were merged using PEAR v0.9.10 with default settings (8), and then sequences were selected by SeqKit v0.8.0 with the parameters -m 245 and -M 260 (9). Selection of operational taxonomic units (OTUs) and assignment of appropriate taxa were performed using a closed-reference OTU-picking script in QIIME v1.9.1 (10) and the SILVA database (release 132) with 97% identity (11). Relative abundances of representative OTUs (>1%), summed at the family level, are shown in Fig. 1, and the indices to assess diversity were calculated with all of the selected high-quality reads for samples by QIIME v1.9.1 (11) (Table 1).

**TABLE 1:** Summary of 16S rRNA gene amplicon profiles of microbiota in bioreactors^*a*^

Analysis measure	Data by sample:		
	SKMS_3 denitrification reactor	SKMS_5 denitrification reactor	SKMS_6 denitrification reactor
SRA run no.	DRR186126–DRR186128	DRR186123–DRR186125	DRR186120–DRR186122
Reactor vol (liters)	0.78	2.34	2.34
PBSA amt (kg)	1	3	3
Feeding frequency	Twice per day	Twice in 2 days	Twice per day
Breeding water temp (°C)	14–16	14–16	14–16
Sampling date	2 October 2018	2 October 2018	2 October 2018
Diversity			
Estimated sample coverage	0.99	0.99	0.99
No. of OTUs	356.3	673.0	901.7
Shannon diversity	2.80	4.18	3.94
Simpson diversity	0.70	0.82	0.78
Chao1 estimator	662.8	1,129.8	1,475.7
ACE^*b*^ estimator	653.5	1,181.9	1,459.1
No. of high-quality reads	92,893	133,017	213,907

a Polybutylene succinate adipate (PBSA)-packed denitrification reactors.

b ACE, abundance-based coverage estimator.

A total of 439,817 high-quality reads were finally obtained from the three analyzed samples. Prominent microorganisms were classified into the families *Nocardiaceae*, *Chitinophagaceae*, *Xanthobacteraceae*, *Burkholderiaceae*, *Rhodocyclaceae*, *Pseudomonadaceae*, *Rhodanobacteraceae*, and *Xanthomonadaceae*. These data provide basic microbial information to achieve efficient operation of the PBSA denitrification reactor for water treatment of land-based RAS.

### Data availability.

The 16S rRNA gene sequence data set has been deposited in the NCBI Sequence Read Archive (SRA) under accession number DRP005307 and SRA run accession numbers DRR186120 to DRR186128.
